# Treatment of displaced intra-articular calcaneus fractures: a current concepts review

**DOI:** 10.1051/sicotj/2017044

**Published:** 2017-10-16

**Authors:** Mandeep S. Dhillon, Sharad Prabhakar

**Affiliations:** 1 Department of Orthopaedics, Post Graduate Institute of Medical Education and Research Sector 12 Chandigarh 160012 India

**Keywords:** Displaced intra-articular calcaneus fractures, Current concepts review

## Abstract

Displaced Intra-Articular Calcaneus fractures (DIACFs) represent a source of tremendous disability to the patient, economic burden to the society and a treatment challenge to the average orthopaedic surgeon. To date, no single approach is universally applicable to all calcaneus fractures. Despite a plethora of published meta-analyses and recent randomized controlled trials, the literature is still unclear and offers conflicting recommendations. The aim of this current concepts review is to assess the latest available data and offer pragmatic and practical recommendations to address some of the issues surrounding DIACFs.

## Introduction

Displaced Intra-Articular Calcaneus fractures (DIACFs) represent a source of potential disability to the patient, economic burden to the society and a treatment challenge to the average orthopaedic surgeon. To date, no single approach is universally applicable to all calcaneus fractures [1]. The goal of treatment has been accurate anatomic reduction, stable fixation with the aim of early functional rehabilitation while avoiding potentially devastating soft tissue complications [2, 3]. It is also well established that pre-existing co-morbidities such as peripheral vascular disease, diabetes and smoking adversely affect wound healing following open reduction and internal fixation (ORIF) of calcaneus fractures [4, 5]. However, despite several published meta-analyses and recent randomized controlled trials, the literature is still unclear and offers conflicting recommendations [1, 2, 4]. The aim of this current concepts review is to sift through the data and offer pragmatic recommendations to address some of the issues surrounding DIACFs.

## Operative versus nonoperative treatment

It has been well established that patients with DIACFs have poorer functional results than those for other orthopaedic conditions [6]. Buckley et al. [2] in their randomized controlled trial in 2002 stated that without stratification of the groups, the functional results of treatment of DIACFs operative or nonoperatively were similar. However, it was pointed out by the authors that women, younger patients (< 29 yrs old), those not receiving workers’ compensation, lighter workload, anatomical reduction or a step-off < 2 mm after surgical reduction had significantly better functional scores following surgery.

Over the past ten years a large number of randomized controlled trials have been conducted by various authors [2, 3, 5, 7–14] in an attempt to determine the efficacy of open reduction and fixation of calcaneus fractures versus nonoperative treatment (Table 1). However, given the varying sample sizes and periods of follow-up, no definite conclusions can be drawn. While authors like Agren et al. [5], Bahari Kashani et al. [8], Nouraei and Moosa [9] and Dooley et al. [12] supported operative intervention with selected indications, others reported equivocal findings [10, 11, 15].

**Table 1.** Overview of existing randomized controlled trials on operative vs nonoperative treatment of calcaneus fractures.

Author, Year	Findings
Griffin et al. 2014 [7]	UK HeFt trial. Operative treatment compared with nonoperative care showed no symptomatic or functional advantage. Risk of complications was higher after surgery. Stated that operative treatment by open reduction and internal fixation is not recommended for these fractures.
Agren et al. 2013 [5]	42 operative group, 40 nonoperative. Operative treatment was not superior in managing displaced intra-articular calcaneal fractures at one year of follow-up. Appeared to have some benefits at eight to twelve years. Operative treatment was associated with a higher risk of complications but a reduced prevalence of posttraumatic arthritis.
Bahari et al. 2013 [8]	84 operative group, 56 nonoperative group. Stated that surgical treatment is the method of choice. Fewer complications in operative group.
Nouraei and Moosa 2011 [9]	31 operative, 30 nonoperative. Open reduction and internal fixation of displaced calcaneal fractures in the absence of open fracture, severe osteoporosis, or comminution, poor general condition may be the preferred method of treatment.
Sharma and Dogra 2011 [10]	15 operative, 15 nonoperative. No significant difference in outcomes.
Ibrahim et al. 2007 [11]	15-year follow-up of displaced intra-articular calcaneal fractures from a randomized controlled trial of conservative versus operative treatment published in 1993. 15 operative, 11 nonoperative. No significant difference in outcomes.
Dooley et al. 2004 [12]	23 operative, 24 nonoperative. Do not definitively support primary operative intervention for bilateral calcaneal fractures.
Howard et al. 2003 [3]	226 operative, 233 nonoperative. Outcome scores in this study tend to support ORIF for calcaneal fractures. ORIF patients are more likely to develop complications.
Buckley et al. 2002 [2]	Without stratification of the groups, the functional results after nonoperative care of displaced intra-articular calcaneal fractures were equivalent to those after operative care. After removal of the patients who were receiving Workers’ compensation, the outcomes were significantly better in some groups of surgically treated patients.
Rodriguez-Merchan et al. 1999 [13]	28 operative, 30 nonoperative. Results better in surgically treated patients.
Thordarson and Krieger 1996 [14]	15 operative, 11 nonoperative. Operative treatment had superior results.
Parmar et al. 1993 [15]	25 operative, 31 nonoperative. No significant difference.

A number of meta-analyses [16–26] have also been published on displaced intra-articular calcaneal fractures (Table 2). However, almost all studies cite insufficient evidence to make a recommendation. Others like Zhang et al. [16], Luo et al. [17] and Liu et al. [18] have stated that surgical interventions in the hands of experienced surgeons have better outcomes and less subtalar fusions are subsequently required.

**Table 2.** Overview of meta-analysis on displaced intra-articular calcaneus fractures.

Author, Year	Patients pooled, Conclusions
Zhang et al. 2016 [16]	908 patients. Surgical outcomes are based on experience. Improvement in gait and shoe wear after surgery.
Luo et al. 2016 [17]	824 patients. Less subtalar fusions but more complications after ORIF. Insufficient evidence for recommendations.
Liu et al. 2015 [18]	966 patients. Surgery protects against subtalar arthrodesis.
Dhillon and Gahlot 2014 [19]	703 patients. Insufficient evidence.
Bruce and Sutherland 2013 [20]	602 patients. Insufficient evidence.
Jiang et al. 2012 [21]	891 patients. Surgery is probably the optimal choice.
Gogoulias et al. 2009 [22]	611 patients. Insufficient evidence.
Bondi et al. 2007 [23]	557 patients. Not possible to draw conclusions.
Bajammal et al. 2005 [24]	534 patients. Insufficient evidence.
Randle et al. 2000 [25]	242 patients. Patients with operative intervention tend to return to work earlier.
Bridgman et al. 2000 [26]	134 patients. Insufficient evidence.

Bruce and Sutherland in their Cochrane review [20] published in 2013 stated that there was insufficient high-quality evidence to establish whether surgical or nonoperative treatment is better for DIACFs. The authors did mention however that the Buckley et al. [2] trial formed a large part of the review. Furthermore, the majority of the procedures (73%) were conducted by a single experienced surgeon and once the workers’ compensation cases were excluded, surgical outcomes improved in subgroup analysis. They also suggested that subgroup analysis was conducted and presented across multiple publications [27–33], resulting in different sample sizes, time to follow-up and reanalysis of primary outcomes. The authors also mentioned that data from the then just concluded multicentric UK Heel Fracture Trial (HeFt) was eagerly awaited.

The UK Heel Fracture trial (HeFt) by Griffin et al. [7] was published in July 2014 as a pragmatic, multicentric, two-arm, parallel group, assessor blinded randomized controlled trial with the sensational claim that operative treatment compared with nonoperative care showed no symptomatic or functional advantage after two years in patients with DIACFs, and the risk of complications was higher after surgery. Thus ORIF was not recommended for calcaneus fractures.

The trial was severely criticized by several researchers for faulty methodology. Gandhi et al. [34] stated that the “baby had been thrown out with the bathwater”. Pearce et al. [35] pointed out that selection bias was a key determining factor. Only 502 of 2006 patients with calcaneal fractures were found eligible for randomization in this study. Furthermore, only 151 of 502 patients agreed to take part in the study, representing a meager 7.5% of all of the calcaneal fractures attending the centres involved in the study. Twenty-seven surgeons in 22 different hospitals operated on a median of only two fractures for this study. Most of the severely displaced fractures requiring surgical treatment had been excluded from the study.

Buckley et al. [36], in a commentary in response to the study in 2015, reiterated that younger patients with simple DIACFs and without workers’ compensation do well surgically. DIACFs need to be managed by specialty surgeons. Nonoperative treatment of DIACFs leads to more subtalar fusions. Future trends point towards limited open reductions with small incisions and a lower risk of complications.

## Extensile versus minimally invasive approaches

The conventional extensile lateral approach remains the “gold standard” to which other minimally invasive approaches are compared [37]. Minimally invasive techniques (MIS) have been devised to overcome the wound complications associated with the conventional lateral approach [1]. MIS refers to a plethora of limited incision approaches or percutaneous fixation relying on indirect reduction techniques under image intensification or even arthroscopy assisted [38, 39]. A recent systematic review by van Hoeve and Poeze in 2016 [40] states that percutaneous reduction and screw osteosynthesis and minimally invasive open techniques resulted in significantly better outcomes compared with external fixation and other techniques. Out of the 46 studies included in the review, covering 2018 calcaneus fractures, only one study by Chen et al. [41] was a randomized controlled trial comparing outcomes between percutaneous screw fixation and cementing or conventional ORIF in calcaneus fractures. A recent randomized controlled trial published by Khurana et al. [42] stated that an extensile approach in a tertiary care hospital, in the hands of an experienced surgeon, has better outcomes. However, the MIS group in the study was a heterogenous group including both percutaneous and limited open approaches. Thus there is paucity of high-quality evidence in the literature to make any firm recommendations.

Minimally invasive approaches are therefore considered to be ideal for patients with compromised skin conditions, associated comorbidities like diabetes or smoking. However, the interventions are limited by the fact that reductions are easier to achieve earlier within two weeks [1].

## Primary subtalar arthrodesis

It has been argued that in Sanders type IV fractures, articular comminution and cartilage injury are severe enough to preclude satisfactory joint reduction and thus primary ORIF and subtalar arthrodesis is an option [1]. Buckley et al. [43] in 2014 in a small randomized controlled trial were unable to demonstrate a significant difference in outcomes between ORIF alone and ORIF with subtalar fusion for Sanders type IV fractures. However, the authors stated that a primary fusion may decrease time away from work and may be economically beneficial.

Dhillon et al. [44] responded to Buckley et al.’s results stating that advocating primary fusion in Sanders IV type fractures was incorrect since only 25% of patients would eventually need fusion. Furthermore, patients with subtalar fracture dislocations, bilateral Sanders IV were not assessed for primary fusion.

Currently, there is no clear evidence to refute or favour primary subtalar arthrodesis.

## The myth of the “constant fragment”

The soft tissue attachments, along with the interosseous talocalcaneal ligaments which bind the sustentaculum to the talus, have historically led to the sustentaculum being described as the “constant fragment” [45]. Traditionally ORIF via a lateral approach involved reducing the lateral fragments to the sustentaculum tali fragment. Berberian et al. in 2013 [46] retrospectively reviewed the computed tomography (CT) scans of 88 patients with 100 DIACFs for evidence of sustentacular displacement and found that the fragment was displaced in 40% of fractures. Gitajin et al. in 2014 [47] reported similar results in 20 % of DIACFs. However currently, there is no data to evaluate a combined medial and lateral approach and its effect on the functional outcome.

## Conclusion

DIACFs are injuries which demand specialist intervention. RCTs and inconclusive meta-analysis underscore the fact that no single approach can be applied as a generalization to all calcaneal fractures. Operative intervention in defined subsets with anatomical reduction and meticulous soft tissue dissection yields favourable outcomes. Minimally invasive or limited open approaches, either image intensifier or arthroscopy assisted, have shown promising results. In all, a thorough evaluation of the patient and his comorbidities, the fracture pattern and soft tissue coverage and the surgeon and his level of skill and experience are required before deciding a line of treatment.

## Conflict of interest

The authors declare that they have no conflict of interest in relation with this paper.
